# 利用全基因组光学图谱检测多发性骨髓瘤细胞遗传学异常

**DOI:** 10.3760/cma.j.cn121090-20230915-00123

**Published:** 2024-03

**Authors:** 艳芳 王, 朕豪 张, 化 王, 连永 郤, 菲 董, 红梅 景

**Affiliations:** 北京大学第三医院血液内科，北京 100191 Department of Hematology, Peking University Third Hospital, Beijing 100191, China

## Abstract

多发性骨髓瘤（MM）是一种以单克隆浆细胞在骨髓中异常增殖为特征的血液肿瘤，MM细胞具有复杂的染色体数目异常和结构异常，这些异常对于患者的危险度分层以及疗效评估具有重要的意义。全基因组光学图谱（OGM）是一项新型的细胞遗传学检测技术，目前在肿瘤、辅助生殖、发育异常等领域已经显示出一定的应用前景，本研究首次利用OGM技术对5例新发MM患者的细胞遗传学异常进行检测，并将其与传统细胞遗传学检测技术进行了比较，从而评估该技术对染色体异常的检出能力以及在MM中的临床应用价值。

## Abstract (en)

Multiple myeloma (MM) is a plasma cell neoplasm characterized by numerous chromosomal number and structural abnormalities, which are of great significance for risk stratification and response evaluation of MM patients. Optical genome mapping (OGM) is a novel technology that has the potential to resolve many of the issues and limitations associated with traditional cytogenetic methods. To date, the clinical utility of OGM has been validated in the fields of cancer, reproduction, and embryonic dysplasia, et al. In this study, we compared OGM to traditional techniques for the first time in five newly diagnosed MM patients, and evaluated the potential of OGM for detecting cytogenetic aberrations and its clinical application value in MM.

多发性骨髓瘤（MM）是一种以单克隆浆细胞在骨髓中异常增殖为特征的血液肿瘤，其发生率约占所有血液肿瘤的10％[1]。虽然新药的应用在一定程度上提高了MM患者的生存率，但是复发仍然是不可避免的问题，这可能与MM细胞高度异质性以及克隆演化有一定关系[2]。MM具有复杂的细胞遗传学异常，包括染色体的数目异常和结构变异（SV），它们不仅参与MM的发生、发展，而且在MM的预后分层体系中占有重要地位[3]–[4]。

全基因组光学图谱（OGM）是一项新型的细胞遗传学检测技术，它能够在基因组范围检测多种类型的染色体平衡/非平衡性SV以及拷贝数变异（CNV），分辨率低至500 bp，平均读长200 kb，对于分析超长以及复杂SV具有独特的优势，在肿瘤、辅助生殖、发育异常等领域已经显示出一定的应用前景[5]–[7]。研究发现，将OGM技术用于白血病患者可以提高传统细胞遗传学方法的检出能力，识别出新的遗传学异常，对于改善白血病的诊断以及亚型分类是一个潜在的新工具[8]–[11]。本研究首次利用OGM技术对5例新发MM患者的细胞遗传学异常进行了检测，并将其结果与染色体核型、FISH以及芯片结果进行了全面比较，从而评估OGM 技术在MM中的临床应用价值。

## 病例与方法

1. 病例：本研究纳入2022年8月至2022年12月在北京大学第三医院就诊的初诊MM患者5例，诊断标准参照王建祥主编的《血液系统疾病诊疗规范》[12]，其中男4例，女1例，中位年龄58（47～64）岁；骨髓中原始幼稚浆细胞比例均>50.0％，范围 50.5％～80.0％。该研究经北京大学第三医院伦理委员会批准（批件号：M2021665），并获得患者的知情同意。

2. 染色体核型分析：将4 ml肝素抗凝骨髓样本加入培养基中，另添加GM-CSF联合IL-6作为刺激剂，对骨髓细胞进行24 h培养，常规制片、G显带进行核型分析，每例患者分析20个中期细胞。根据《人类细胞基因组学国际命名体系（ISCN）（2020）》进行核型描述。

3. 染色体FISH检测：应用磁珠分选技术对骨髓标本中CD138^+^浆细胞进行富集，D13S319、RB1、P53、CKS1B（1q21）/CDKN2C（1p32）、IgH、IgH/CCND1、IgH/FGFR3、IgH/MAF、IgH/MAFB、C-MYC探针均购自北京金菩嘉医疗科技有限公司。将CD138^+^浆细胞与相应探针杂交，经洗片、复染后，在荧光显微镜下观察荧光信号，每个探针至少分析200个间期细胞。

4. 染色体微阵列芯片检测：提取骨髓样本基因组DNA，应用Affymetrix CytoScan 750K芯片对样本进行全基因组CNV分析。该芯片包含20万个单核苷酸多态性（SNP）探针以及55万个非多态性CNV探针，平均分辨率为50 kb，以上芯片杂交及数据分析由上海韦翰斯生物医药科技有限公司完成。

5. 染色体OGM检测：提取骨髓样本中超长基因组DNA，使用Bionano DLS试剂盒对DNA进行荧光标记和骨架染色，将标记好的DNA加载到Saphy半导体芯片，进行高分辨成像，应用Bionano Solve v3.5.1软件对数据进行组装，并与内置参考基因组（hg38）进行比对。数据分析包括两套流程（pipeline）：一个为SV pipeline，能够检测插入（insertion）/缺失（deletion）（≥500 bp）,重复（duplication）（≥30 kb），倒位（inversion）（≥30 kb），易位（translocation）（≥50 kb）；另一个为CNV pipeline，主要检测较大片段的拷贝数获得/丢失（gain/loss）（≥5 Mb）以及非整倍体（aneuploidie），以上检测及数据分析由上海韦翰斯生物医药科技有限公司完成。

6. 统计学处理：采用SPSS 22.0统计软件进行分析，OGM与芯片两种方法检出CNV数目的比较采用配对*t*检验，*P*<0.05为差异具有统计学意义。

## 结果

1. 染色体核型分析结果：5例患者均分析了20个中期分裂象，其中有2例检出染色体异常，而且均表现为复杂核型（表1）；其余3例患者染色体正常，其中1例患者出现14号染色体随体增大，核型为46, XY, 14ps+[20]，随体增大通常为体质性改变，多见于老年男性。

**表1:** 5例多发性骨髓瘤患者的核型分析、FISH以及微阵列芯片结果

例号	核型	FISH	微阵列芯片
1	46, XX[20]	del(17p), IgH/CCND1	gain(5p/5q,7q,9p/9q, 10q, 11p/11q, 15p/15q,19p,21p/21q, Xq), loss(1p,16p,17p,Xq)
2	46, XY,14ps+[20]	del(13q), gain(1q21), IgH	gain(1q,5p/5q,7p/7q,9p/9q,15q,19p/19q,21p/21q), loss(4p/4q, 8p, 12q, 13p/13q,14q,19p)
3	46, XY[20]	del(13q), del(17p), del(IgH)	loss(13q, 14q, 17p)
4	39-42, XY, t(3;8)(p14;q24), add(3)(p13),-4, add(8)(q11), t(8;11)(q24.3;q13), −13,−14,−17, inv(18)(q21.1q22), +19,−20,−21, add(22)(q13)[cp16]/46,XY[4]	del(13q), del(17p), del(IgH)	gain(3p, 11q, 17q, Xp), loss(13q, 14q, 17p, Xp)
5	44,XY,+1,der(1;21)(q10;q10)×2, inv(2)(p13q31),−6, t(8;14)(q24.1;q32), der(14)t(6;14)(p21;q32), der(16)t(1;16)(q21;q13),+21,−22[9]/46,XY[11]	gain(1q21), IgH, C-MYC	gain(1q, Yp), loss(1p, 16q, 22q)

2. 染色体FISH检测结果：5例患者均检出特异性染色体异常（表1），其中13q（D13S319和RB1基因）缺失3例；17p（P53基因）缺失3例；1q21扩增2例；IgH重排3例，C-MYC重排1例；进一步利用IgH易位探针检测3例IgH重排的伙伴基因，结果显示例1的 IgH/CCND1融合基因阳性，例2和例5的4种常见的IgH融合基因均为阴性，考虑例5 C-MYC重排阳性，分析例5可能发生了IgH/MYC易位。

3. 染色体微阵列芯片检测结果：5例患者芯片均检出大量的CNV，具体累及的染色体区域见表1。5例患者CNV检出数目分别为46个（gain 14个，loss 32个）、69个（gain 22个，loss 47个）、27个（gain 0个，loss 27个）、52个（gain 10个，loss 42个）、45个（gain 15个，loss 30个），其中≥5 Mb的CNV分别有5个、21个、0个、1个、14个。

4. 染色体OGM检测结果：5例患者OGM检测平均DNA分子长度为227 kb，有效覆盖深度为370×，5例患者检出的染色体SV数目见表2，具体累及的染色体区域见表3，在染色体上的分布见图1。

**表2:** 5例多发性骨髓瘤患者全基因组光学图谱检出不同类型结构变异的数目

结构变异	例1	例2	例3	例4	例5
插入	3	10	10	9	16
缺失	40	53	38	57	22
倒位	3	0	0	0	4
重复	14	13	0	11	13
染色体内易位	7	3	0	7	2
染色体间易位	9	2	0	5	4
拷贝数获得	4	17	0	4	6
拷贝数丢失	1	4	0	1	11
非整倍体获得	5	5	0	1	1
非整倍体丢失	0	2	0	5	1

总计	86	109	48	100	80

**表3:** 5例多发性骨髓瘤患者全基因组光学图谱检出的染色体异常

例号	全基因组光学图谱检出染色体异常
1	ins(1p, 5q, 11p), del(1p, 3p, 6q, 7q, 13q, 14q, 16p, 17p, Xq), inv(11q, Xq), dup(7q, 10q, 16q, 19p, 22q, Xq), fus(1p, 11q, 19q), t(1;X), t(1;6), t(6;8), t(7;8), t(7;X), t(11;14), t(19;X), gain(11p/11q, 21p/21q), loss(1p), +5, +9, +11, +15, +21
2	ins(4p, 4q, 5p, 5q, 9p, 10p, 12q, 17q, Xp), del(3q, 4p, 5q, 8p, 12q, 14q, 19p, Xq), dup(12q, 13q), fus(8p, 19p, 19q), t(8;19), t(13;14), gain(1q, 5p/5q,7p/7q,9p/9q, 15q, 19p/19q,21p/21q), loss(4p/4q,12q,13p/13q),-4,+5, +7,+9,-13,+19,+21
3	ins(2q, 4q, 7q, 10p, 10q, 16q), del(4q, 7q, 8q, 13q, 14q, 17q)
4	ins(2q, 5q, 6p, 6q, 11p, 12q, 13q, 15q), del(3p, 5q, 14q, 17p, 19q, 20q, Xq), dup(11q, 17q, Xp), gain(3p, 8q), loss(13q), fus(14q, 17q), t(3;8), t(8;11), t(11;18), t(17;22), t(19;20), −4, −13, −14, −17, +19, −21
5	ins(2p, 5p, 5q, 14q, 15q, 17p), del(1p, 5q, 6p, 10q, 11q, 14q, 16q), inv(15q), dup(1q, Yp), gain(1q), loss(1p, 16q, 22q), fus(6q, 10q), t(1;5),t(6;14), t(8;14), +21, −22

**图1:**
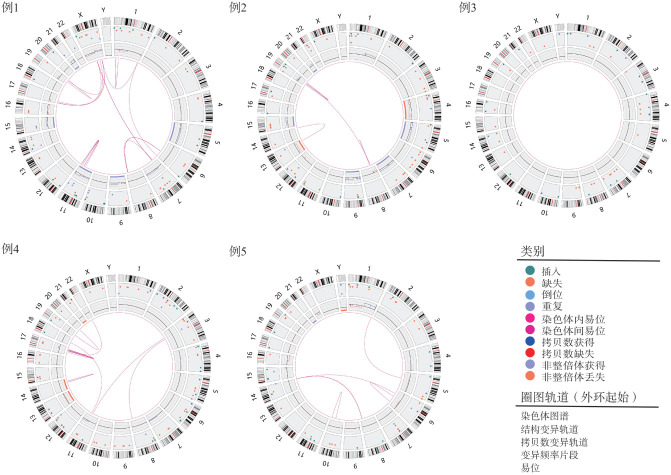
5例多发性骨髓瘤患者染色体结构变异分布圈图

例1、例2、例3均为正常核型，无法与OGM进行比对；例4核型共检出13个染色体异常，7个数目异常中有6个被OGM检出，6个结构异常中有4个被OGM检出，77％（10/13）的核型结果与OGM一致；例5核型共检出9个染色体异常，其中4个数目异常中有2个被OGM检出，5个结构异常中有3个被OGM检出，56％（5/9）的核型结果与OGM一致。

5例患者FISH检出的染色体异常均被OGM检出，根据OGM结果，例2 IgH重排是发生了t（13;14）（q32.1;q32.33）染色体易位（图2A）；例5 OGM检出t（6;14）（p21.1;q32.33）和t（8;14）（q24.21;q32.33）两个染色体易位（图2B），与核型结果一致，也证实了FISH结果中该患者形成了IgH/MYC融合基因。

**图2:**
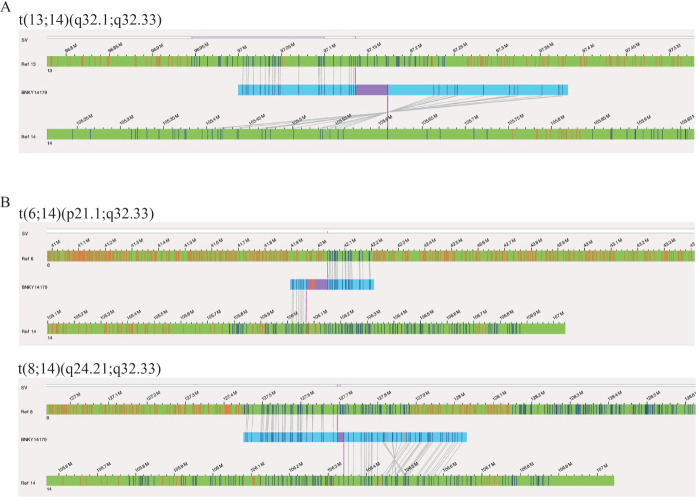
2例多发性骨髓瘤患者IgH易位OGM基因图谱 A 例2 OGM检出t（13;14）（q32.1;q32.33）染色体易位；B 例5 OGM检出t（6;14）（p21.1;q32.33）和t（8;14）（q24.21;q32.33）两个染色体易位 注 OGM：全基因组光学图谱

5例患者芯片检出的CNV均被OGM检出，OGM两套分析流程的分辨率不同，在分析拷贝数异常时应包括重复、缺失、获得以及丢失（表2）。5例患者OGM检出CNV的总数分别为59个、87个、38个、73个、52个，明显多于芯片的CNV检出数目（*P*＝0.005）；对于≥5 Mb的CNV，OGM（gain/loss）分别检出5个、21个、0个、5个、17个，与芯片检出数目差异无统计学意义（*P*>0.05）。

## 讨论

骨髓瘤细胞基因组高度不稳定，具有复杂的染色体数目和结构异常，目前，细胞遗传学特征，如gain（1q21）、del（17p）、t（4;14）等已成为高危MM的主要评价指标；另外，MM细胞中的克隆或亚克隆性遗传学异常可以在药物的选择性压力下发生演化，从而影响患者的治疗反应和生存时间[2]–[4]，因此，对MM患者进行全面的遗传学筛查，有助于更好的了解MM患者在治疗和预后上的异质性。

OGM技术具有超长片段、高分辨率以及全基因组范围分析的特点。Neveling等[8]对52例血液肿瘤患者进行了OGM以及核型、FISH、CNV芯片检测，结果显示OGM技术的敏感性为100％，SV和CNV的阳性预测值分别为96％和81％，对于核型分析<5个异常的样本，所有临床报告的异常均被OGM检出；对于复杂核型（≥5个异常）样本，OGM与传统检测技术结果大体一致，但由于分辨率较高，OGM的结果更为复杂。Lestringant等[9]对10例ALL样本进行检测，结果显示利用传统检测技术发现的80个异常中，72个（90％）被OGM检出，但同时OGM发现12个额外的异常。Suttorp等[10]对24例儿童AML患者进行检测，虽然70％（17/24）的OGM结果与核型分析不完全一致，但两种方法对预后评估具有较好的一致性（95％），这可能与两种方法的检测结果均为复杂异常或均检出高危遗传学异常有关；另外，OGM可以作为白血病微小残留病监测的新的标志物。

本研究首次利用OGM技术对5例初诊MM患者结构变异进行了分析，因为当骨髓涂片中原幼浆细胞比例超过50％时，磁珠分选与不分选FISH结果之间差异无统计学意义[13]，为保证足够样本量，本实验中芯片和OGM检测未进行浆细胞富集。研究结果显示OGM不仅与FISH和芯片结果具有较好的一致性，而且还能发现更多额外的染色体异常，但是与核型分析结果仍有一定差异。本研究5例患者中有3例均为正常核型，但利用其他检测方法均检出染色体异常，这就表明MM核型分析容易受骨髓瘤细胞增殖分裂的影响，发生漏检；例4和例5核型结果中分别有23％和44％的异常没有被OGM检出，这些差异结果正确与否还有待证实，如例4核型发现add（22q），但OGM和芯片均未检出累及22号染色体的拷贝数增加，由于核型分辨率较低，对于染色体微小片段的扩增或缺失检测能力有限，因此核型中add（22q）可能有误；另外，例5核型结果中t（1;21）（q10;q10）染色体易位未被OGM检出，因为OGM是利用酶切、标记的方法进行检测，大量复杂重复序列可能会干扰酶切位点，对于发生在具有复杂重复序列，如着丝粒、端粒的断裂可能会存在漏检[14]。由于芯片不能检测平衡易位，而OGM作为一种新型的染色体结构异常检测工具，对于平衡易位的检出能力还需要通过其他方法进一步验证。

OGM较芯片分辨率高，能够识别低至500 bp左右的染色体微小片段的异常。本研究显示OGM检出的CNV总数明显多于芯片，而且芯片不能直接检出非整倍体；对于≥5 Mb的较大片段的CNV，两种检测技术的一致性较好。由于本研究例数较少，OGM检出的大量CNV有何临床意义尚不清楚，今后还需要继续扩大样本量，从而为临床筛选潜在的预后标志物以及肿瘤MRD监测标志物。

综上所述，OGM较传统的细胞遗传学技术相比，能够一次获得更加全面的遗传学信息，节约临床样本，降低检测成本；而且无需中期分裂相，不受MM细胞体外培养增殖率低的影响，在MM中具有一定的临床应用价值，这些遗传学信息有可能为MM患者进一步危险度分层，评估疾病复发或进展风险提供更多的实验室依据。
